# Cortico-muscular connectivity is modulated by passive and active Lokomat-assisted Gait

**DOI:** 10.1038/s41598-023-48072-x

**Published:** 2023-12-07

**Authors:** Fiorenzo Artoni, Andrea Cometa, Stefania Dalise, Valentina Azzollini, Silvestro Micera, Carmelo Chisari

**Affiliations:** 1 Department of Clinical Neurosciences, University of Genève, Faculty of Medicine, 1211 Geneva, Switzerland; 2 The BioRobotics Institute and Department of Excellence in Robotics and AI, Scuola Superiore Sant’Anna, Viale Rinaldo Piaggio 34, 56025 Pontedera, Italy; 3 University School for Advanced Studies IUSS Pavia, 27100 Pavia, Italy; 4 Unit of Neurorehabilitation, Pisa University Hospital, Pisa, Italy; 5 Department of Translational Research and New Technologies in Medicine and Surgery, University of Pisa, Pisa, Italy; 6 Translational Neural Engineering Laboratory (TNE), École Polytechnique Fédérale de Lausanne, Campus Biotech, Geneva, Switzerland; 7 Ago Neurotechnologies Sàrl, 1201 Geneva, Switzerland

**Keywords:** Neuroscience, Motor control, Characterization and analytical techniques

## Abstract

The effects of robotic-assisted gait (RAG) training, besides conventional therapy, on neuroplasticity mechanisms and cortical integration in locomotion are still uncertain. To advance our knowledge on the matter, we determined the involvement of motor cortical areas in the control of muscle activity in healthy subjects, during RAG with Lokomat, both with maximal guidance force (100 GF—passive RAG) and without guidance force (0 GF—active RAG) as customary in rehabilitation treatments. We applied a novel cortico-muscular connectivity estimation procedure, based on Partial Directed Coherence, to jointly study source localized EEG and EMG activity during rest (standing) and active/passive RAG. We found greater cortico-cortical connectivity, with higher path length and tendency toward segregation during rest than in both RAG conditions, for all frequency bands except for delta. We also found higher cortico-muscular connectivity in distal muscles during swing (0 GF), and stance (100 GF), highlighting the importance of direct supraspinal control to maintain balance, even when gait is supported by a robotic exoskeleton. Source-localized connectivity shows that this control is driven mainly by the parietal and frontal lobes. The involvement of many cortical areas also in passive RAG (100 GF) justifies the use of the 100 GF RAG training for neurorehabilitation, with the aim of enhancing cortical-muscle connections and driving neural plasticity in neurological patients.

## Introduction

In recent years, there has been a surge in research focused on the development of robotic devices for rehabilitation. This has led to their widespread adoption in the field of neurorehabilitation^1^.

The need to establish standardized guidelines for the appropriate use of robotic-assisted therapy in clinical practice has been a subject of extensive debate. The recent Italian consensus conference known as CICERONE, a unique initiative of its kind on a global scale, has played a significant role in this discussion^1^. The Consensus synthesized evidence on the effectiveness of robotic-assisted therapy in treating neurological conditions, suggesting the setting of application for specific disabling conditions.

Robotic-assisted therapy is particularly recommended for post-stroke patients to enhance lower limb motor function, including gait and strength, ultimately fostering greater independence. Robotic-assisted gait (RAG) training has thus become widely popular in the rehabilitation field, because of the robotic systems’ capacity to yield high-intensity training, as it allows a high number of task-oriented repetitions, and the possibility of reproducing and assessing the kinematics during the gait cycle^2–4^.

Experience-dependent RAG can enhance neuroplasticity and motor re-learning^5^, however, scientific evidence of its superiority over conventional therapy and its effect on neuroplasticity mechanisms and cortical integration in locomotion are still under discussion^6–8^.

**Figure 1:**
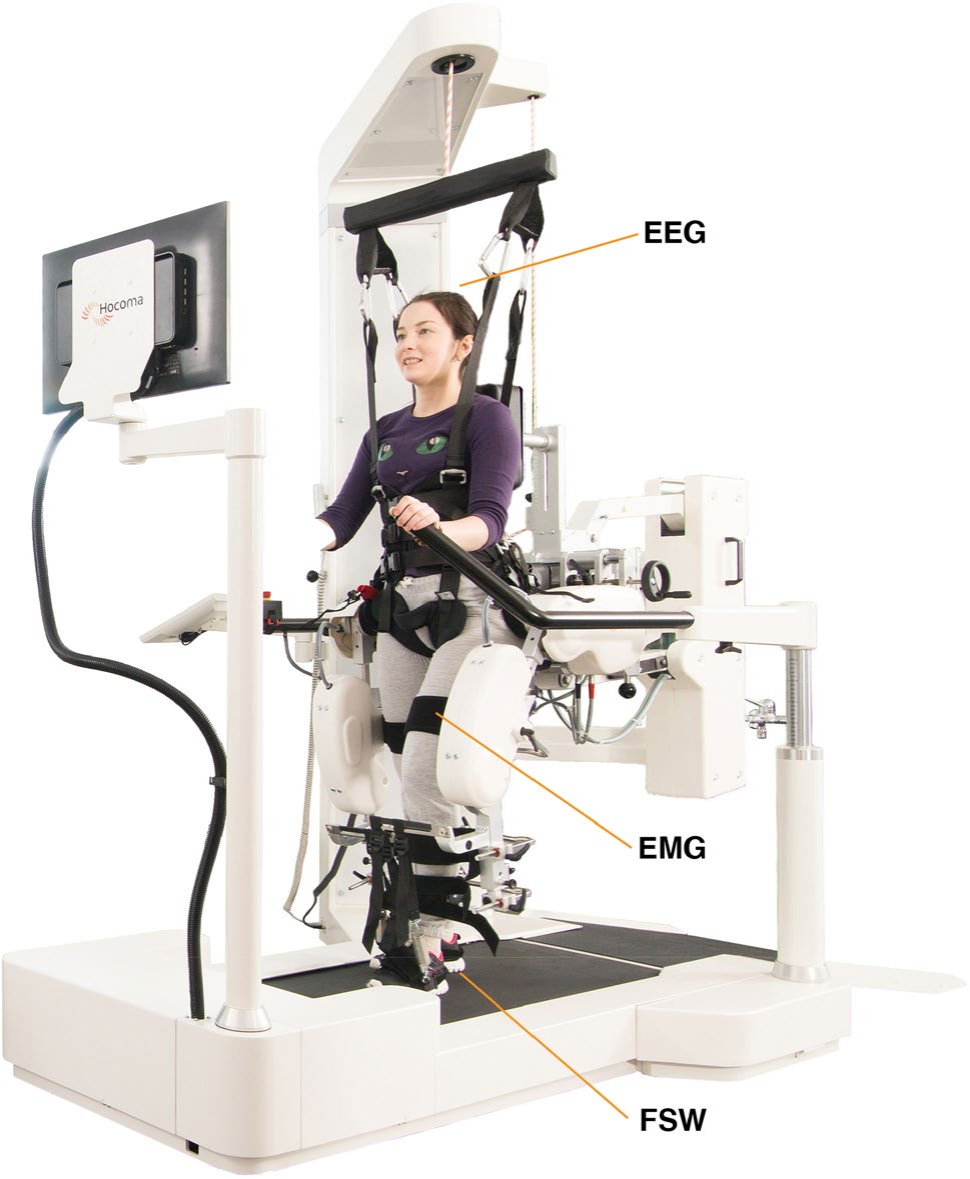
Lokomat exoskeleton. Figure representing the robotic device used. In this study, subjects are supported by a harness providing up to full body weight support while performing gait training. The exoskeleton was used both actively (0% guidance force support) and passively (100% guidance force support). While using the exoskeleton, 4 footswitches (FSW), 6 bipolar wireless EMG electrodes and a 64 channels EEG cap were allowed to image the brain/body activity during the gait tasks. Figure adapted from the website https://www.hocoma.com.

One of the most utilized devices for RAG training is the Lokomat (Hokoma, Switzerland). This robotic system incorporates motorized components along with a bodyweight-supported treadmill (Fig. 1). During the training sessions, the device provides both body-weight-support and guidance force (GF), which assists the patient in following a physiological gait pattern. These parameters can be modified to tailor the treatment and adapt it to the patient’s performance, thus allowing a partial personalization of the treatment plan^9^.

The Lokomat has been shown to be efficient in improving balance, trunk control, and gait^7,10–12^. Nonetheless, the precise mechanism underlying these improvements remains a subject of inquiry. It remains uncertain whether the observed enhancements stem from peripheral musculoskeletal and spinal mechanisms or whether RAG training improves connectivity and brain-muscle integration.

Human walking is a complex process, involving not only spinal interneuronal networks^13–16^ but necessitating also the integration of supraspinal motor commands^17^, to maintain balance^18^, perform overground walking^19^, and, intriguingly, stereotyped treadmill walking^20,21^. Wagner et al.^22^ showed significant modulations of midline sensorimotor mu and beta rhythms by different levels of participation (i.e., active and passive modes) in robotic-assisted treadmill walking. Also, in a previous study of ours, we provided evidence of cortical activity associated with locomotion and identified a significant causal unidirectional drive from the contralateral motor cortex to limb muscles during the swing phase of treadmill gait. These insights overturned the traditional view that the human cortex has a limited role in the control of stereotyped locomotion and suggested new hypotheses about the mechanisms underlying gait under other conditions^20^.

The aim of this study is to determine the engagement of cortical motor regions in the control of leg muscles activity during a session of RAG with Lokomat. The study particularly explores two opposite conditions widely used in rehabilitation settings: maximum (100%) and null (0%) GF, in healthy individuals. Our hypothesis is that cortico-muscular connectivity patterns can be observed and influenced by RAG, even in the passive modalities (i.e., under maximum GF). To this aim, we applied a novel connectivity estimation procedure, based on Partial Directed Connectivity (PDC), to jointly study source localized EEG and EMG activity during rest (standing) and active/passive RAG.

After a global connectivity analysis, we focused our attention on brain regions implicated in gait motor execution and control, supporting the idea of supraspinal involvement in the process of walking. The purpose was to shed light on the roles played by distinct brain areas in different gait conditions. Mapping the cortico-muscular connectivity during RAG can provide an indication on whether RAG training can facilitate gait restoration through the reorganization of the central neural system, rather than just improving gait at an automatic-functional level.

## Materials and methods

### Experimental protocol

To determine the involvement of cortical motor areas in the control of leg muscle activity, we simultaneously recorded EEG and EMG data of 6 lower-limb muscles (Vastus Medialis—VM, Biceps Femoris—BF, Tibialis Anterior—TA), bilaterally from 10 healthy subjects (31 ± 4 years old) during RAG. The chosen muscles play an essential role in maintaining balance and facilitating normal gait movements. Specifically, the quadriceps, represented here by the Vastus Medialis, are responsible for knee extension during the stance phase, which ensures leg stability when bearing weight. During the forward swing phase of the leg, the hamstrings (represented by the Biceps Femoris) come into play, as they flex the knee, aiding in limb clearance. Additionally, the Tibialis Anterior muscle contributes by dorsiflexing the foot, preparing it for ground contact^23^. The subjects were asked to walk within the Hocoma Lokomat Exoskeleton at 2 km/h. All the subjects provided informed consent prior to participation in this study. This study was approved by the ethics committee of the University of Pisa (Prot. 0105605, 28/2023). All research was performed in accordance with the Declaration of Helsinki and all relevant guidelines/regulation. The experiment protocol consisted of a 3-min resting period while standing within the Lokomat, followed by 2 walking sessions of 6 min each, respectively with (100 GF) and without (0 GF) GF-support. During the task, subjects were instructed to look ahead at a fixed point on a screen in front of them and to maintain, as far as possible, a relaxed gait.

### Signals recording

**Figure 2:**
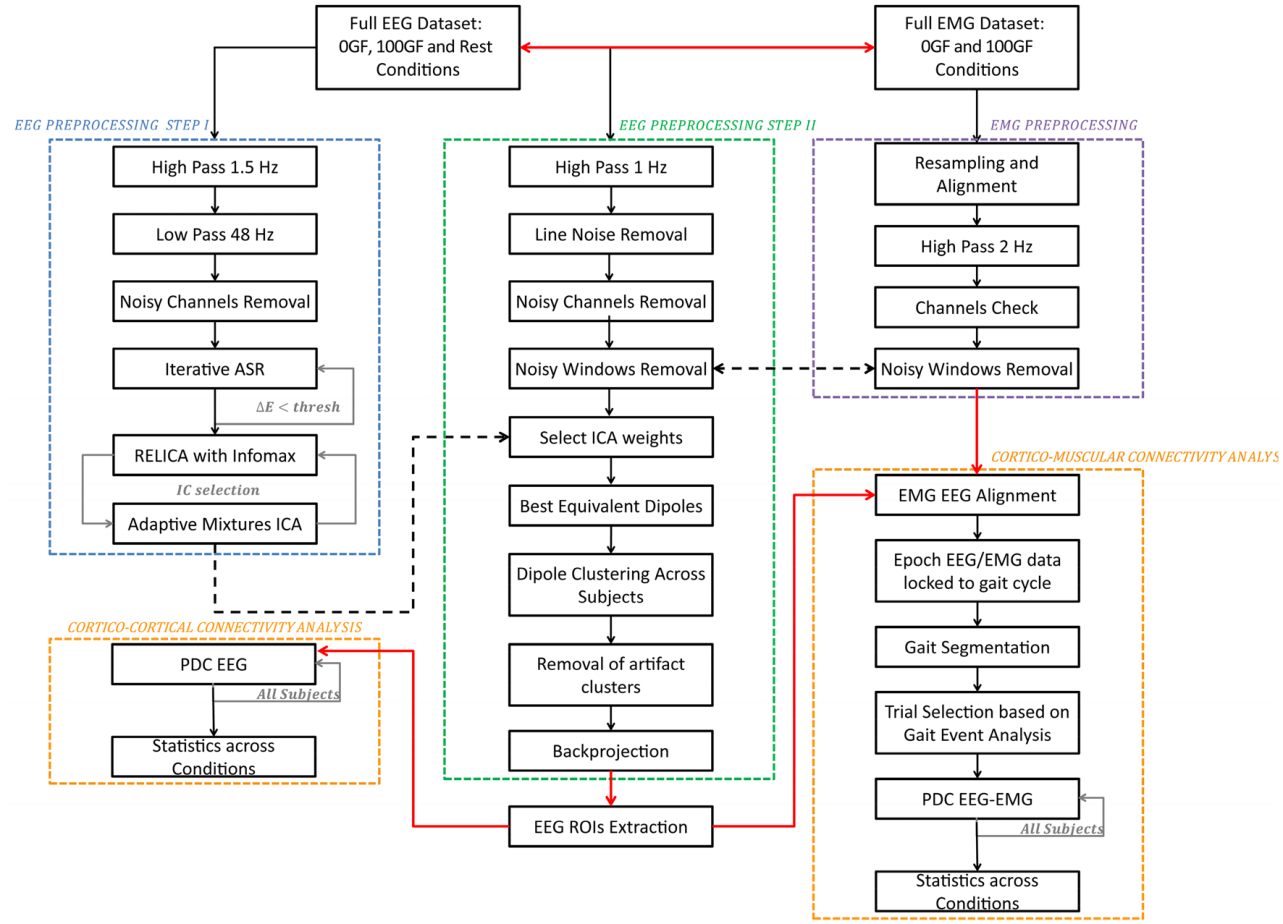
Full process pipeline. The EEG data underwent two preprocessing steps, namely EEG Preprocessing Step I (blue box) and EEG Preprocessing Step II (green box). Independent Components (ICs) were extracted within Step I. IC weights were then transferred to the dataset, conservatively processed according to Step II. Step I is designed to extract ICs with the best possible reliability. Step II is more conservative, and it is designed to retain the maximum amount of information for subsequent connectivity analyses. Each IC was source-localized using a dipolar model, and artifact clusters of ICs were removed from the data. The backprojected EEG data was then used to perform distributed source localization, and the AAL atlas EEG ROIs time courses were extracted. Cortico-cortical connectivity analysis was then performed across all subjects for 0 GF, 100 GF, and Rest conditions using the time course of the source-localized activity within the regions of interest (ROIs) extracted after Step II. In parallel, EMG data were aligned to EEG, high-passed at 2 Hz and noisy windows labeled for rejection. Only time windows that were not labeled as artifact either for EEG or EMG were retained. Cortico-muscular connectivity (assessed through Partial Directed Coherence—PDC) was estimated for 0 GF and 100 GF respectively on epoched data, time locked to the gait cycle (see “Methods” for more details). Group-level statistical analysis across all subjects and conditions was then performed.

The 64-channel EEG data were recorded at a sampling rate of 2048 Hz using a custom active electrode cap (actiCAP, Brain Products, GmbH, Germany), linked to a Micromed amplifier (SD MRI), certified for clinical use. The electrode montage was compliant to the 5% International 10/20 System^24^. Careful scalp preparation was conducted to ensure a 20 kΩ impedance in at least 95% of derivations both at the beginning and the end of recordings. Re-gelling was performed as needed. EMG data was simultaneously recorded with a wireless EMG system (BTS Free EMG 300) at a sampling rate of 1000 Hz. Synchronization with measured jitter below 2 ms was ensured by following the procedures described in^25^. Four footswitches (FSW) were attached to the heel and forefoot of the shoe soles and acquired without relative delay or jitter by the same EMG amplifier. Bipolar wireless EMG electrodes were positioned by expert clinicians according to internationally-recognized guidelines (SENIAM—www.seniam.org) on 3 muscles of each leg, namely Biceps Femoris (BF), Vastus Medialis (VM), Tibialis Anterior (TA). The full processing procedure is outlined in Fig. 2 and comprises two EEG preprocessing steps (blue and green boxes), one EMG preprocessing step (violet box) and cortico-cortical and cortico-muscular connectivity analysis steps (orange box).

### EEG preprocessing step 1

The EEG data of standing and walking conditions were concatenated and were first high-pass filtered with a zero-phase 1.5-Hz cutoff, 24th order maximally-flat Chebyshev type II inverse filter to remove slow drifts. Particular care was taken to ensure that filters did not cross boundaries (i.e., gaps across conditions). Data were then low-pass filtered using a zero-phase 48 Hz 70th order Chevyshev type II filter to remove high-frequency noise and resampled to 512 Hz. Channels with prolonged prominent artifacts were removed based on a combination of methods, including visual inspection, kurtosis (above 95th percentile of the distribution calculated after preliminarily removing extreme artifacts, i.e. with amplitudes above 300 mV), and the “*clean_rawdata*” EEGLAB plugin. Remaining channels were average re-referenced and submitted to the artifact subspace reconstruction procedure (ASR)^26^, using the standing-up resting data for calibration, to remove high-amplitude artifacts from the EEG recorded during walking. Using an approach already introduced and used in previous works to reduce the impact of outliers^20,27^, ASR was iteratively applied using a 20 std deviations threshold, until the amount of modified data was below 5%. RELICA with AMICA core^28^ was then used to decompose data into Independent Components (ICs). Within AMICA, rejection of data whose likelihood exceeded 5 standard deviations from median likelihood, under the AMICA-derived model, was performed 5 times at 5-iterations intervals. The maximum number of iterations was set to 10.000. Within RELICA, EEG data were bootstrapped 200 times with point-by-point resampling^29^. For each bootstrap repetition, ICs were extracted using CUDAICA^30^, a GPU-supported implementation of the Infomax algorithm^31^. ICs across all repetitions, within subjects, were then clustered according to their mutual similarity using an agglomerative hierarchical clustering method with group average-linkage criterion as agglomeration strategy^28^. The number of clusters was set to be equal to the number of scalp channels reduced by one (to account for average referencing). To avoid the risk of non-convergence of the ICA algorithm and skewing the results, ICA decompositions where more than 10% of ICs could not be separated into different clusters were excluded, and clustering performed again on the remaining ICs^28^. Each cluster size defined the quality index that determined the stability of each IC. As in previous works^20,28,32^, to combine the advantages of RELICA with the accuracy of AMICA decompositions, AMICA ICs (extracted on non-bootstrapped data) were paired to RELICA IC clusters based on the absolute value of the correlation coefficients between their time courses. The size of the cluster associated to each IC indicated its quality index, which, coupled with a measure of dipolarity (i.e., residual variance of the fitted equivalent dipole–dipfit algorithm within EEGLAB) allowed to determine the reliability of each IC. Only reliable components (both stable and dipolar, and therefore not a result of inadequate IC convergence) could be tagged as artifacts in subsequent processing steps. The evaluation of the reliability of ICs (15 ICs with Quality Index above 95% and dipolarity above 85% on average, per subject) allows to confirm/evaluate the appropriateness of preprocessing steps such as the removal of movement and other movement and non-brain artifacts from the EEG data.

### EEG preprocessing step 2

To minimize the removal of brain activity while maximizing the quality of the ICA decomposition, final ICA weights resulting from Preprocessing Step 1 were applied to data more conservatively preprocessed within EEG Preprocessing Step 2 (see green box in Fig. 2). Raw data were high-pass filtered with a zero-phase 1.0 Hz, 24th order Chebyshev type 2 filter, line noise was further filtered with a 50-Hz comb notch filter without real poles^33^. Bad channels and epochs containing high-amplitude artifacts, high frequency noise and other irregular artifacts, already identified in EEG Preprocessing Step 1, were removed. Only stable ICs (i.e., with quality index above 95%) were considered and categorized as physiological artifacts, non-physiological artifacts or brain-related activity based on the Tailarach coordinates of their best fitting equivalent dipole, their power-spectra, time-course and time–frequency transforms. ICs for each subject were clustered across subjects via EEGLAB routines^34^ using distance vectors combining differences in dipole location, power spectral density (1–45 Hz), and the scalp projection pattern (scalp map) for each IC. The dimensionality of the resulting joint vector was reduced to fifteen principal components by principal component analysis (PCA). Vectors were clustered using k-means (k = 15). Components further than three standard deviations from any of the resulting cluster centers (outlier ICs) were relegated to a separate “outlier” cluster. Clusters related to neck-muscle artifacts, ocular activity, heart, line noise and mechanical artifacts were removed.

### EMG preprocessing

EMG data were high-pass filtered using a zero-phase, 1 Hz, Chebyshev type II inverse 24th-order filter and the EMG channel signals were checked for drifts, discontinuities, and flatline periods. To maintain perfect EEG-EMG alignment, artifact-laden EMG epochs (e.g., due to electrode displacement, loss of contact) were removed also from the EEG dataset and viceversa.

### EEG source imaging and ROIs extraction

Underlying brain source signals were determined by applying low resolution electromagnetic tomography—LORETA^35^ within Cartool^36^ on IC-reconstructed scalp EEG data. The lead field for the inverse solution was calculated for 64 electrode positions and the average brain of the Montreal Neurological Institute (MNI) in a grey matter constrained head model using the LSMAC head model with 6000 distributed solution points^37^. Activation biases were reduced by standardizing across time and each solution point the estimated current densities of each participant. The solution of the inverse problem yields estimates of continuous time courses for cortical sources. Cortical regions of interest (ROI) were defined according to the Automated Anatomical Labelling Atlas 3—AAL3^38^. Each ROI source signal was computed by averaging estimated cortical source activities across the source space ROI voxels defined by the atlas. We focused the analyses on the following ROIs defined for each cortical hemisphere: Parietal Lobe—postcentral gyrus, superior parietal gyrus, inferior parietal gyrus, supramarginal gyrus, angular gyrus, precuneus; Frontal Lobe—precentral gyrus, Rolandic operculum, supplementary motor area; Posterior Fossa. The choice of these specific regions of interest (ROIs) stems from their established significance in gait-related functions. The chosen brain areas have been extensively associated with various aspects of motor control, balance, and cognitive processes pertinent to walking^39,40^.

### Connectivity estimation

We estimated cortico-cortical connectivity and cortico-muscular connectivity using the partial directed coherence (PDC)^41^. PDC is a frequency-dependent connectivity measure based on the Geweke–Granger causality framework^42,43^.

The advantage of PDC lies in its unique capability to differentiate between cortico-muscular connectivity and muscular-cortico connectivity, a distinction that eludes traditionally employed techniques like coherence. This distinctive feature allows to precisely unravel the directional influences between cortical regions and specific muscles. However, it's important to note that PDC assumes linearity, which may limit its accuracy in capturing non-linear interactions.

Within the Granger causality framework, a time series ${\mathrm{x}}_{\mathrm{j}}(\mathrm{t})$ causes another time series ${\mathrm{x}}_{\mathrm{i}}(\mathrm{t})$ if knowledge of past samples of ${\mathrm{x}}_{\mathrm{j}}(\mathrm{t})$ reduces the prediction error for the current sample of ${\mathrm{x}}_{\mathrm{i}}(\mathrm{t})$^44^. The relationship between the time series can be described by a multivariate autoregressive (MVAR) model on $\mathrm{X}(\mathrm{t})$:

1 $$\mathrm{X}\left(\mathrm{t}\right)={[{\mathrm{x}}_{1}\left(\mathrm{t}\right), {\mathrm{x}}_{2}\left(\mathrm{t}\right),\dots ,{\mathrm{x}}_{\mathrm{D}}\left(\mathrm{t}\right)]}^{\mathrm{T}}$$

where D is the total number of channels.

The MVAR model assumes a linear relationship between channels in $\mathrm{X}(\mathrm{t})$ of the form:

2 $$\mathrm{X}\left(\mathrm{t}\right)=\sum_{\mathrm{k}=1}^{\mathrm{p}}{\mathrm{a}}_{\mathrm{k}}\mathrm{X}\left(\mathrm{t}-\mathrm{k}\right)+\mathrm{e}(\mathrm{t})$$

where a_k_ are the $\mathrm{DxD}$ MVAR coefficient matrices, $\mathrm{e}\left(\mathrm{t}\right)$ is a white noise process and p is the model order.

The a_k_ matrices were estimated using the ordinary least squares algorithm^45^. The model order p was determined using the Bayesian Information Criterion^44,46^. Equation (2) can be rewritten in frequency domain as $\mathrm{X}\left(\mathrm{f}\right)\mathrm{A}\left(\mathrm{f}\right)=\mathrm{E}(\mathrm{f})$, where

3 $$\mathrm{A}\left(\mathrm{f}\right)=\mathrm{ I}- \sum_{\mathrm{k}=0}^{\mathrm{p}}{\mathrm{A}}_{\mathrm{k}}{\mathrm{e}}^{-\mathrm{j}2\mathrm{\pi fk}}$$

From $\mathrm{A}\left(\mathrm{f}\right)$, the PDC describing the directional causality from time series ${\mathrm{x}}_{\mathrm{j}}(\mathrm{t})$ to time series ${\mathrm{x}}_{\mathrm{i}}(\mathrm{t})$ can be defined as:

4 $${\mathrm{c}}_{\mathrm{ij}}^{2}\left(\mathrm{f}\right) = \frac{{\left|{\mathrm{A}}_{\mathrm{ij}}\left(\mathrm{f}\right)\right|}^{2}}{\sqrt{\sum_{\mathrm{l}=1}^{\mathrm{D}}{\left|{\mathrm{A}}_{\mathrm{lj}}\left(\mathrm{f}\right)\right|}^{2}}}$$

where ${\mathrm{A}}_{\mathrm{ij}}\left(\mathrm{f}\right)$ is the element at the ith row and jth column of the matrix $\mathrm{A}\left(\mathrm{f}\right)$. The connectivity in a certain frequency band [f_1_, f_2_] can be obtained by averaging the PDC values from ${\mathrm{c}}_{\mathrm{ij}}^{2}\left({\mathrm{f}}_{1}\right)$ to ${\mathrm{c}}_{\mathrm{ij}}^{2}\left({\mathrm{f}}_{2}\right)$.

### Network analysis

The PDC values of the cortico-cortical connectivity can be seen as edges of a network composed by a collection of nodes (ROIs). In the following network analysis, PDC values computed between all possible pairs of ROIs were used to form weighted networks for each subject and frequency band separately.

We computed network features that summarize the segregation and integration of a network. Segregation is the tendency of nodes to be organized in clusters and is measured by the average clustering coefficient. Integration is the capacity of the network to exchange information and is measured by the characteristic path length.

For weighted networks, such as those estimated by the PDC, the clustering coefficient of a node $i$ is defined as^47^:

5 $${CC}_{i} = \frac{2}{{k}_{i}({k}_{i}-1)}\sum_{j,k}{\left(\widehat{{c}_{ij}}\widehat{{c}_{jk}}\widehat{{c}_{ki}}\right)}^{1/3}$$

where ${k}_{i}$ is the sum of outgoing and incoming connections to node $i$ (i.e., node degree) and $\widehat{{c}_{ij}}$ is the PDC value from node $j$ to node $i$ in a given frequency or frequency band, normalized by the largest weight in the network. The mean clustering coefficient ${C}_{c}$ is computed by averaging the clustering coefficient of all single nodes.

The weighted characteristic path length can be calculated as^48^:

6 $${\mathrm{L}}_{c} = \frac{1}{D}\sum_{i = 1}^{D}\frac{{\sum }_{j = 1, j \ne i}^{D}{\mathrm{d}}_{ij}}{D-1}$$

where

7 $${\mathrm{d}}_{ij} = \sum_{{c}_{uv} \in { g}_{ij}}\frac{1}{{c}_{uv}}$$

is the shortest weighted path length and ${g}_{ij}$ is the shortest path from $j$ to $i.$

We also computed the total connectivity strength (i.e. the sum of all the PDC values between ROIs) and the small worldness. The small worldness is defined as the ratio between the average clustering coefficient and the characteristic path length^49^:

8 $$S = \frac{{C}_{c}}{{L}_{c}}$$

Small worldness measures the property of a network of having high levels of local clustering among nodes and short paths that connect all the nodes.

### Cortico-cortical and cortico-muscular connectivity

We estimated the cortico-cortical connectivity by calculating the PDC values between all ROIs for the three conditions (0 GF, 100 GF and rest) in five different frequency bands: delta (0–4 Hz), theta (4–8 Hz), alpha (8–13 Hz), beta (13–30 Hz), and gamma (30–40 Hz).

To calculate the cortico-muscular connectivity, the EEG and EMG datasets were first epoched. Epochs were extracted for both guidance force levels. Each epoch was extracted from the heel strike of one foot to the heel strike of the other foot, in order to put the swing of the two legs in different epochs. PDC values between ROIs and EMG were calculated separately for each epoch and each frequency and averaged across trials for each subject. Supp. Fig. S1 shows an example of panels highlighting the time/frequency connectivity values between one ROI (Left Angular Gyrus) and muscle (left/right VM). Given their similarity, we averaged connectivity values across frequency bands. We used bootstrap^50^ to compare the directed cortico-muscular connectivity between the two guidance force levels. Confidence intervals for the 0 GF and 100 GF conditions at 95% were extracted from the bootstrap distributions. If the confidence intervals did not overlap, the difference in cortico-muscular connectivity between the guidance force levels was deemed significant.

### Statistical testing

Non-normality of the data was assessed using the Shapiro–Wilk test^51^. Statistical testing was performed using the Wilcoxon signed-rank test^52^ for paired samples and the Mann–Whitney test^53^ for unpaired samples. Non-parametric one-way ANOVA was done using the Kruskal–Wallis test^54^ with Conover post-hocs^55^. Bonferroni correction was used to account for multiple comparisons^56^. Significance was assigned at α = 0.05.

## Results

### Lokomat-assisted treadmill walking affects cortico-cortical connectivity

We computed 3 network-describing features (i.e., the average clustering coefficient, the characteristic path length and the small worldness) on the cortico-cortical connectivity matrices as estimated by the PDC. We calculated these features and the total connectivity strength (i.e., the sum of all the elements of the zero-diagonal connectivity matrix) for each subject, each condition (rest, 0 GF, 100 GF) and each frequency band.

**Figure 3:**
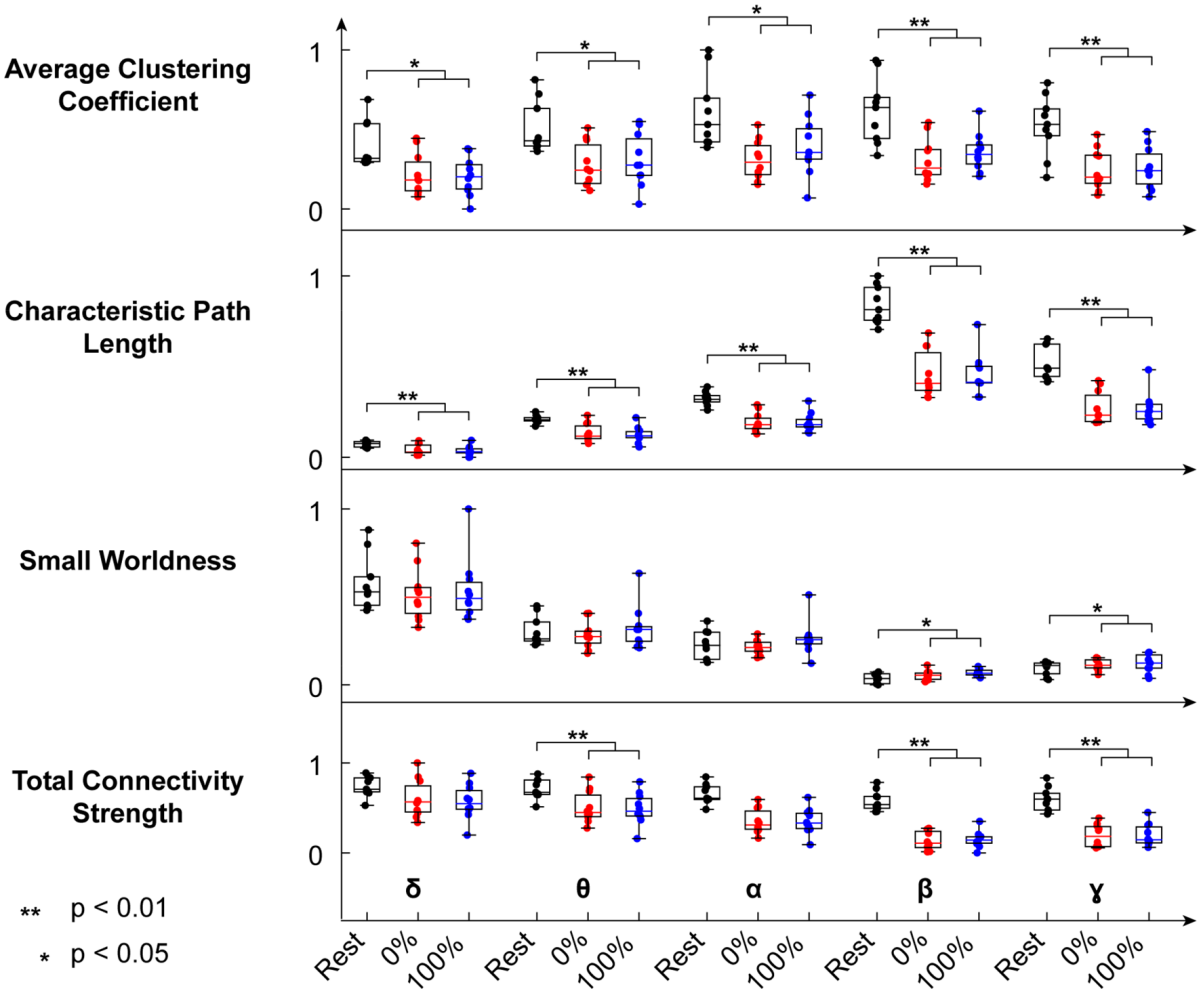
Normalized network features (average path length, small worldness, clustering coefficient and total connectivity strength) of the intracortical connectivity network in the 3 conditions: rest (black), 0% GF (blue) and 100% GF (red).

Differences between rest and walking condition (both 0 GF and 100 GF) were found at all frequencies for the average clustering coefficient and characteristic path length (higher for rest, *p* < 0.05, Wilcoxon signed-rank test with Bonferroni correction). Total connectivity strength was greater during rest than during walking for all frequency bands above delta (*p* < 0.05, Wilcoxon signed-rank test with Bonferroni correction). Finally, small worldness was found to be significantly higher during walking in beta and gamma band (*p* < 0.05, Wilcoxon signed-rank test with Bonferroni correction). No significant differences were found between 0 and 100 GF (Fig. 3).

### Cortico-muscular connectivity differs between 0 and 100 GF

**Figure 4:**
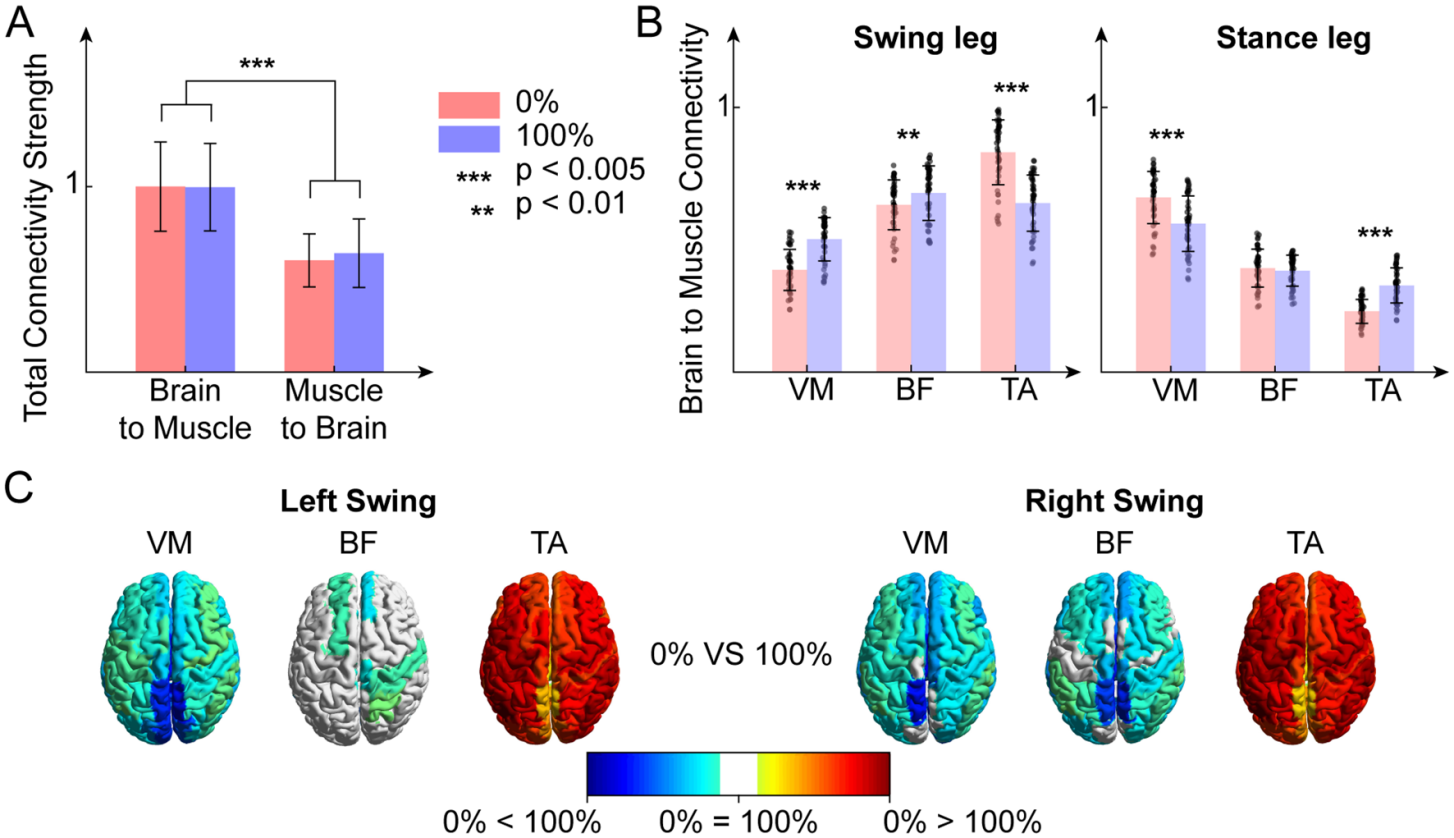
(**A**) Brain-to-muscle normalized connectivity strength and muscle-to-brain normalized connectivity strength for both guidance force levels. (**B**) Normalized brain to muscle connectivity flow toward vastus medialis (VM), tibialis anterior (TA) and biceps femoris (BF) of the swing leg (left) and of the stance leg (right), for both guidance force levels. (**C**) Brain maps of the significative difference in cortico-muscular connectivity between guidance force levels, divided by muscle.

We defined the brain to muscle total connectivity as the sum of the connectivity values from any ROI to any muscle, across trials and subjects. Accordingly, the muscle to brain total connectivity was calculated as the sum of all incoming connections to the cortical ROIs from all the muscles, across trials and subjects. Brain to muscle total connectivity strength was higher than muscle to brain total connectivity strength for both 0 GF and 100 GF conditions (*p* < 0.005, Mann–Whitney test with Bonferroni correction). No difference was found between conditions (Fig. 4A).

In both legs, cortico-muscular connectivity was significantly stronger for 0 GF than 100 GF in distal muscles (TA) during the swing phase (*p* < 0.05, Wilcoxon signed-rank test with Bonferroni correction). On the contrary, for the stance leg, distal muscle cortico-muscle connectivity was lower for 0 GF than 100 GF (Fig. 4B,C).

**Figure 5:**
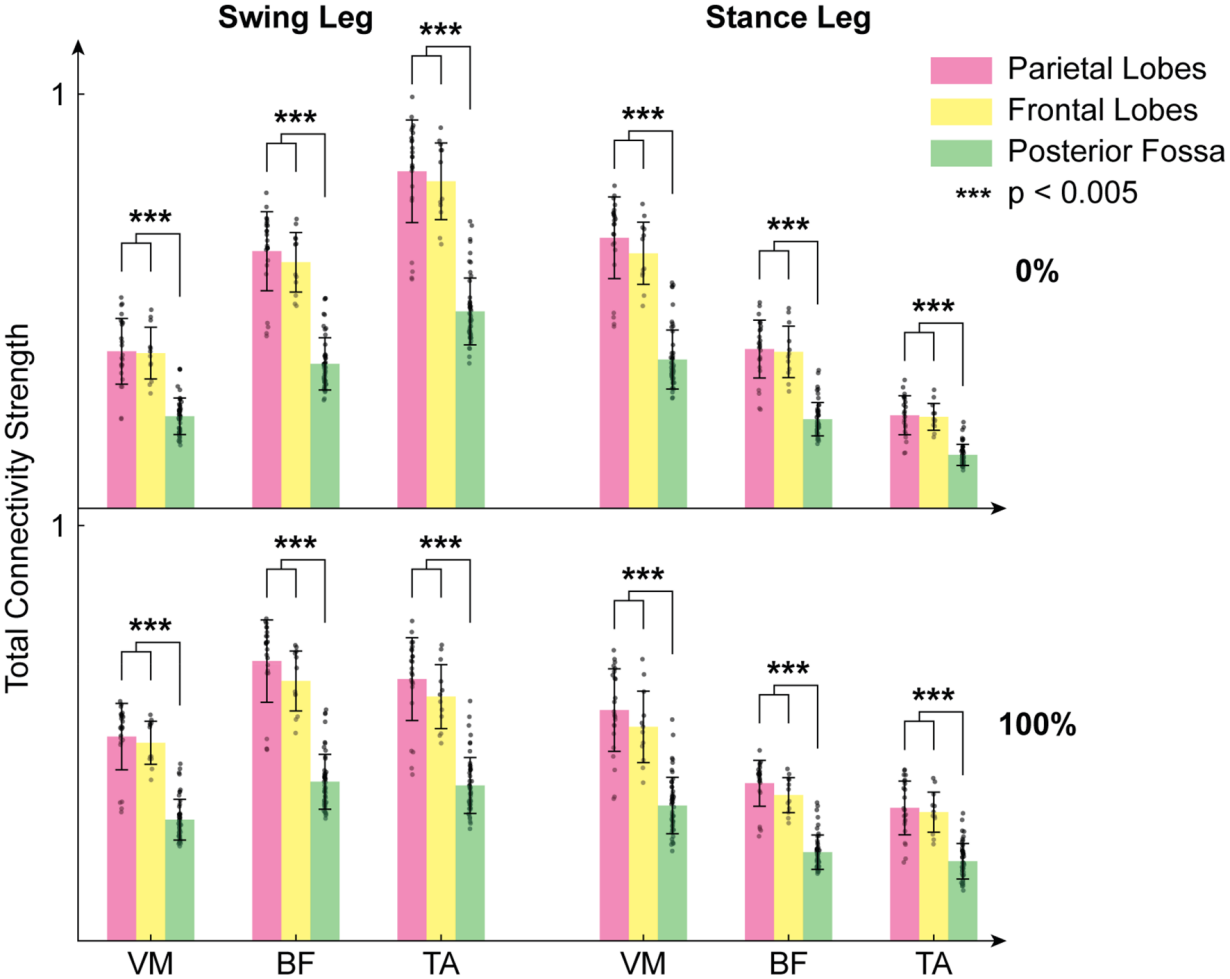
Bar plots of the cortico-muscular connections values directed from the parietal lobes (pink), frontal lobes (yellow), and the posterior fossa (green) to the vastus medialis (VM), tibialis anterior (TA) and biceps femoris (BF) of the swing leg (left) and of the stance leg (right), for both guidance force levels (up: 0% GF, bottom: 100% GF).

We then focused the attention on ROIs involved in motor control and execution in the parietal lobes (precentral gyrus, superior and inferior parietal gyri, supramarginal gyrus, angular gyrus and precuneus), frontal lobes (precentral gyrus, rolandic operculum and supplementary motor area), and posterior fossa. The same global pattern of differences between 0 and 100 GF for the swing leg and stance leg was found locally at the lobe level (Fig. 5). Parietal-driven cortico-muscular connectivity and frontal driven cortico-muscular connectivity were, however, stronger than the connections between the posterior fossa and the muscles (*p* < 0.05, Kruskal–Wallis test with Conover post-hoc).

**Figure 6:**
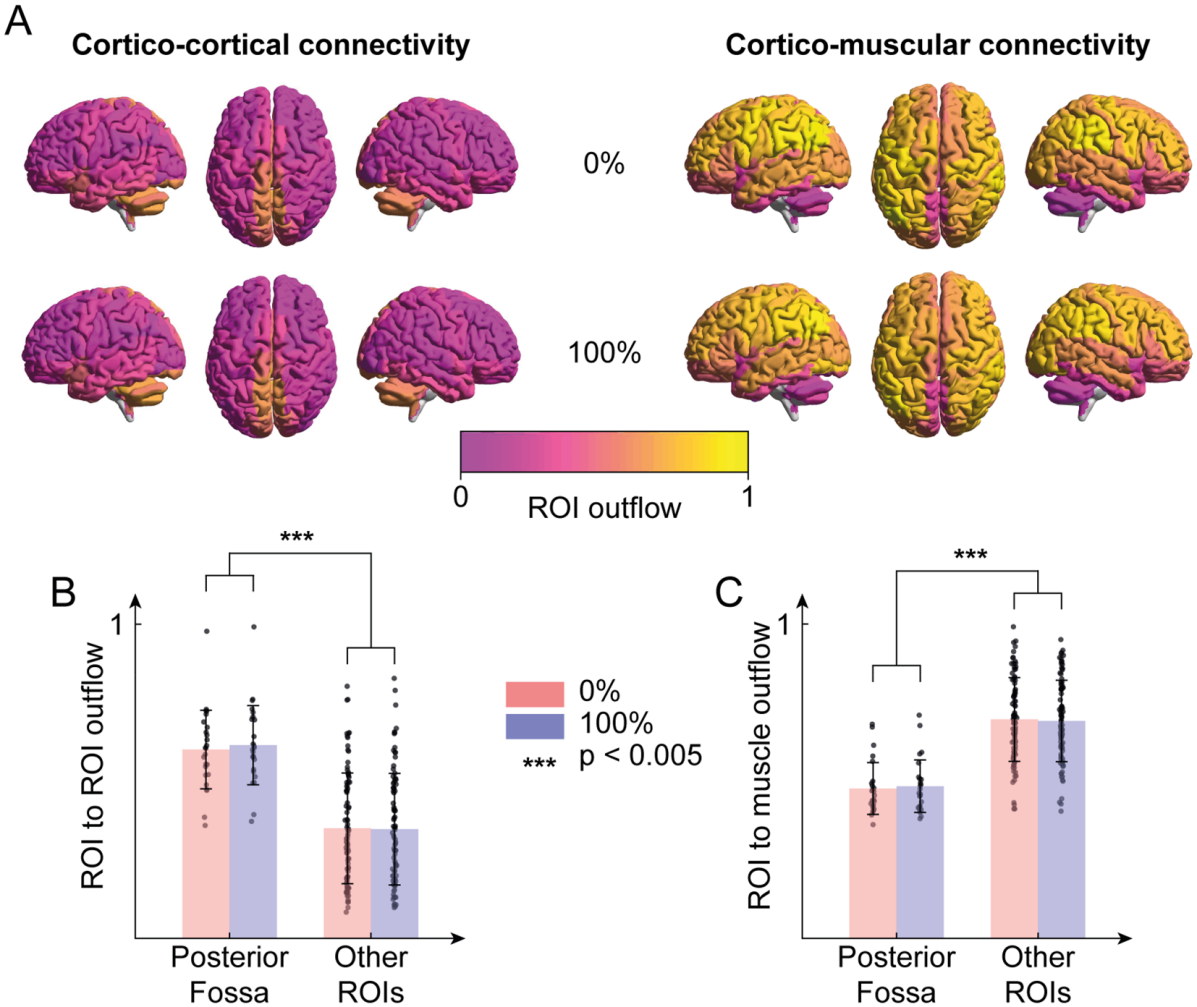
(**A**) Normalized ROI outflow (sum of outcoming connections) in cortico-cortical connectivity in both guidance force levels (left) and normalized ROI outflow in cortico-muscular connectivity in both guidance force levels (right). (**B**) Normalized outflow of the ROIs of the posterior fossa in cortico-cortical connectivity and outflow of all other ROIs, for both guidance force levels. (**C**) Normalized outflow of the ROIs of the posterior fossa in cortico-muscular connectivity and outflow of all other ROIs, for both guidance force levels.

Interestingly, the posterior fossa was found to be a central hub for cortico-cortical connectivity during walking, but with low outflows values in cortico-muscular connectivity (Fig. 6A). Overall, cortico-cortical connectivity arising from the posterior fossa was significantly higher than the connectivity outflow from the ROIs in other brain regions (*p* < 0.005, Mann–Whitney test with Bonferroni correction, Fig. 6B). On the other hand, cortico-muscular connectivity from the ROIs in the posterior fossa was lower than that from the ROIs in other brain regions, not only in the parietal and frontal lobes (*p* < 0.005, Mann–Whitney test with Bonferroni correction, Fig. 6C). Fig. S2 shows that the same results are achieved in the distinct frequency bands (δ to γ).

## Discussion

The widespread use of RAG training in neurorehabilitation highlights its efficacy in improving the functional outcomes in several neurological diseases. However, the most recent reviews have not yet been able to clearly define specific guidelines for optimizing RAG training. This is partially due to a limited understanding of the underlying physiological mechanisms driving the functional improvement. Here we addressed the neurophysiological modifications in healthy subjects during RAG tasks within Lokomat, with the aim to identify the involvement of the cerebral cortex in relation to 3 different conditions: rest, active RAG (0 GF) and passive RAG (100 GF).

Our study shows the involvement of the cortical motor areas in muscle control during both the conditions delivered by the Lokomat system, which highlights the possibility to engage neural outputs with a bottom-up approach even when the movement is completely guided by the device. This is further supported by the absence of statistically significant differences in ROI outflow between the two conditions, which justifies exploring both 0 GF and 100 GF paradigms for neurorehabilitation, with the aim of enhancing cortical-muscle connections.

Our results demonstrated a higher global cortico-cortical connectivity during the rest condition compared to the walking function (both 0% and 100% GF), in particular a higher path length at all the frequencies during rest, showing a stronger total connectivity in all the frequency bands except for delta; on the contrary, during the task we found a greater local connectivity, highlighted by the increased small-worldness index in the beta and gamma bands. The cortical network flexibility between rest and volitional gait is a key feature for lower-limb motor behavior and it is commonly impaired in acquired neurological diseases such as stroke^57^. Surprisingly, we found a connectivity change during both the specific conditions of RAG, highlighting a specialized cortical control of gait even in passive mode. In previous works, cortical-connectivity modifications were described in specific lower limb motor tasks, linked with gait. Spedden et al.^58^ described how the reduction in connectivity strength between the primary motor cortex and the dorsal pre-motor cortex during a lower limb motor task was related to a greater control of ankle dorsiflexion, critical in the swing phase; the same study highlights that the connectivity pattern deviates with aging, suggesting that our results should be interpreted according to an age stratification. Other studies revealed an enhanced functional connectivity only during dual-task walking, considering the cortico-cortical connectivity as a measure of the mental workload^59^, while the involvement of the cortex in rhythmic walking movements is better pointed out by cortico-muscular connectivity measures than cortico-cortical connectivity^20^.

While the presence of a unidirectional brain-to-muscle connectivity during treadmill locomotion has been already demonstrated^20^, in this new study we found that the total brain to muscle connectivity is higher than the muscle to brain connectivity in both the assisted walking conditions. The presence of this pattern in the 100FG condition indicates a persistence of cortical motor control of the lower limb muscles and movement planning in a completely guided condition. This is in accordance to^22^ who found gait-phase related modulations of spectral power during the gait cycle also present during passive walking. In light of this, it appears reasonable that the effectiveness of RAG training on functional improvement might be related to the activation of a central control even when movement is performed in a completely passive mode, which suggests the possibility of inducing neuroplasticity and motor re-learning also in highly-impaired patients.

Furthermore, the two walking conditions (0 GF and 100 GF) have a different cortico-muscular activity pattern during the stance and swing phases of the gait: the cortico-muscular connectivity is higher for the distal muscle (TA) during swing in 0 GF, while it is higher for the proximal muscle (VM) during stance in 100 GF. The results for 0 GF provide a further proof of the importance of the direct supraspinal control in the swing phase^20^ even when gait is supported by the robotic exoskeleton. On the other hand, during stance at 100 GF, supraspinal control might have a preponderant role in stabilizing the proximal grindle, which undergoes a more challenging balance condition. This control is driven mostly by parietal and frontal lobe and less by the posterior fossa.

In fact, it is well known that during walking, cortical areas as premotor, cingulate motor area, supplementary motor and motor areas, cerebellum and basal ganglia actively guide the preparation, planning and execution of movement, both in healthy and in stroke patients^60,61^.

Cortical-muscle connectivity for the posterior fossa was weaker than for parietal and frontal lobes. Yet, in our experimental design, the posterior fossa showed a higher cortico-cortical than cortico-muscular connectivity, compared to other areas. We might speculate that the cerebral areas generating this signal have a central role in coordinating other areas during walking, making the occipital lobes and visual pathway a main hub in this activity. Nonetheless, the signal recorded in this area may be influenced by deeper areas of the posterior fossa of the brain such as the cerebellum, which is widely known to have a key role in the direct locomotor pathway^60^.

It must be noted however that, while the possibility of detecting subcortical electrophysiological activity with high-density EEG source imaging has been recently demonstrated^62^ localizing the sources in these areas without the availability of individual MRI scans is challenging and the spatial resolution of EEG in deep structures remains poor^63^. The site-specific analysis of these areas and the interpretation of their role is beyond the aims of this study, however, in future studies, it would be interesting to develop techniques for a more sophisticated neurophysiological assessment of the role of deep areas such as Cerebellum and Posterior Fossa in gait and gait rehabilitation.

It is worth highlighting that our study reports cortico-muscular *connectivity*, which, as opposed to cortico-muscular *coherence,* allows to explore directional flows of information. Differently from other studies, highlighting cortico-muscular coherence modulations mainly in beta and gamma bands^64–66^ we only report slight differences in cortico-muscular connectivity across frequency bands (Supp Fig. S1). The main reason for this is likely the discrepancy between the spectral range of the main information content for EEG (mainly 1–45 Hz and, in certain cases, up to 100 Hz) and that of sEMG (10–230 Hz and up to 400 Hz or more)^67^. EMG rectification has been used as a way to shift EMG high frequency spectrum towards low frequencies, i.e., < 50 Hz, thus making it comparable to EMG spectral content^68^. Rectification is sometimes followed by a low pass filter or an Hilber Transform step when assessing coherence/connectivity across muscles or extracting muscle synergies^69^. However, EMG preprocessing pipelines and in particular the nonlinear rectification step to study EEG-EMG coherence/connectivity are controversial^68,70–72^. Interesting further studies may focus on analyzing the influence of EMG preprocessing steps on estimating cortico-muscular connectivity.

While gait encompasses a myriad of distinct muscle activation patterns, each tied to the phases of locomotion, we divided gait into two broad phases, namely swing and stance, for several reasons. First, this approach is consistent with the conventional framework of gait analysis research, thus ensuring our findings can resonate with a broader audience, particularly in the field of rehabilitation. Second, classical muscle activation patterns may undergo significant alterations in the context of fully guided gait, as in 100 GF condition, investigated in our study. This deviation from natural gait patterns, introduced by the robotic exoskeleton, could complicate the analysis of muscle-specific activation patterns during the RAG sessions and the comparison between the two conditions. Finally, our system setup allowed to analyse a limited number of muscles per side, preventing a more comprehensive exploration of complex muscle synergies during gait. A more comprehensive experimental setup encompassing a broader array of muscles, might allow us to gain deeper insights into the intricacies of neural control in gait rehabilitation.

## Conclusion

Our results confirm the engagement of the motor cortical regions typically involved in overground and treadmill gait, even in a completely “passive” locomotion, i.e., a RAG session with 100 GF. This suggests the possibility of driving neural plasticity even with a passive use of robotic devices such as Lokomat. Consequently, these results open up the horizon for extending the application of such devices to individuals with significant gait impairments, thereby broadening their clinical relevance.

### Supplementary Information

Supplementary Information.

## Data Availability

The datasets used and/or analysed during the current study available from the corresponding author upon reasonable request.
