# Supplementary material for: Cortico-muscular connectivity is modulated by passive and active Lokomat-assisted Gait
Source: Sci Rep. 2023 Dec 7;13:21618. doi: 10.1038/s41598-023-48072-x (PMC10703891; doi:10.1038/s41598-023-48072-x)
Supplement: Supplementary file 1 — Supplementary Information. [file 41598_2023_48072_MOESM1_ESM.docx]

**Supplementary Materials**

Cortico-Muscular connectivity is modulated by passive and active Lokomat-assisted Gait

Fiorenzo Artoni*^,1,7^, Andrea Cometa^2,3^, Stefania Dalise^4^, Valentina Azzollini^5^, Silvestro Micera^2,6^, Carmelo Chisari^4,5^

**Email:**  * fiorenzo.artoni@unige.ch


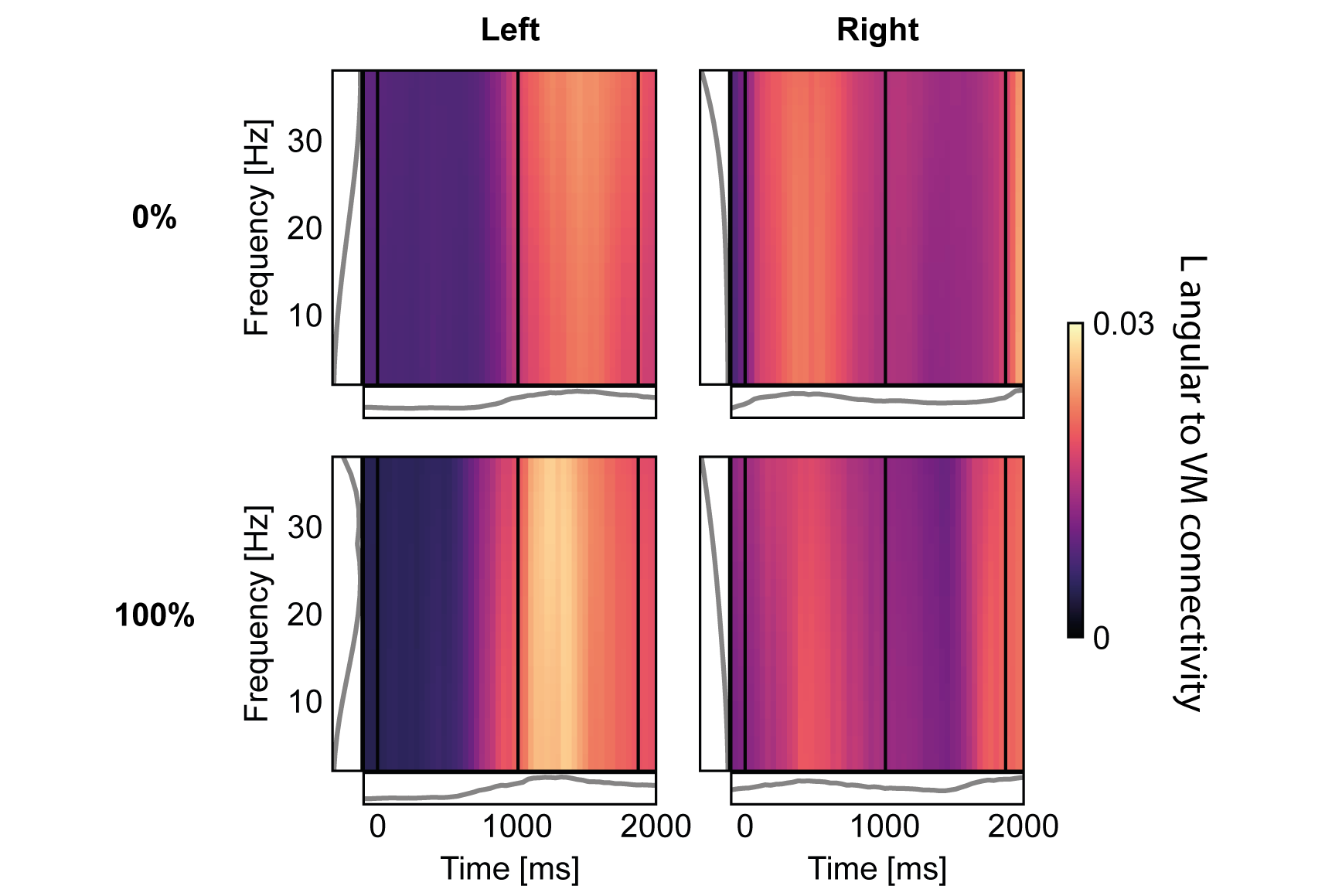


**Supplementary Figure 1. Time-frequency-varying cortico-muscular connectivty.** Time-frequency plots illustrating the cortico-muscular connectivity originating from the left angular gyrus (L angular) to both the left (depicted on the left) and right (depicted on the right) Vasti Mediales (VM) at different guidance force (GF) levels (top: 0% GF, bottom: 100% GF) in a representative subject. Each plot showcases the average cortico-muscular connectivity across all trials. The highest connectivity value for each frequency is indicated on the left of the respective time-frequency plot. The lower plots display the time-dependent average connectivity across all frequencies. In each plot, three vertical black lines mark significant events in the step cycle: the right heel-on, left heel-on, and once again the right heel-on. The interval between the first and second vertical lines corresponds to the swing of the right leg, while the duration between the second and third vertical lines represents the swing of the left leg.


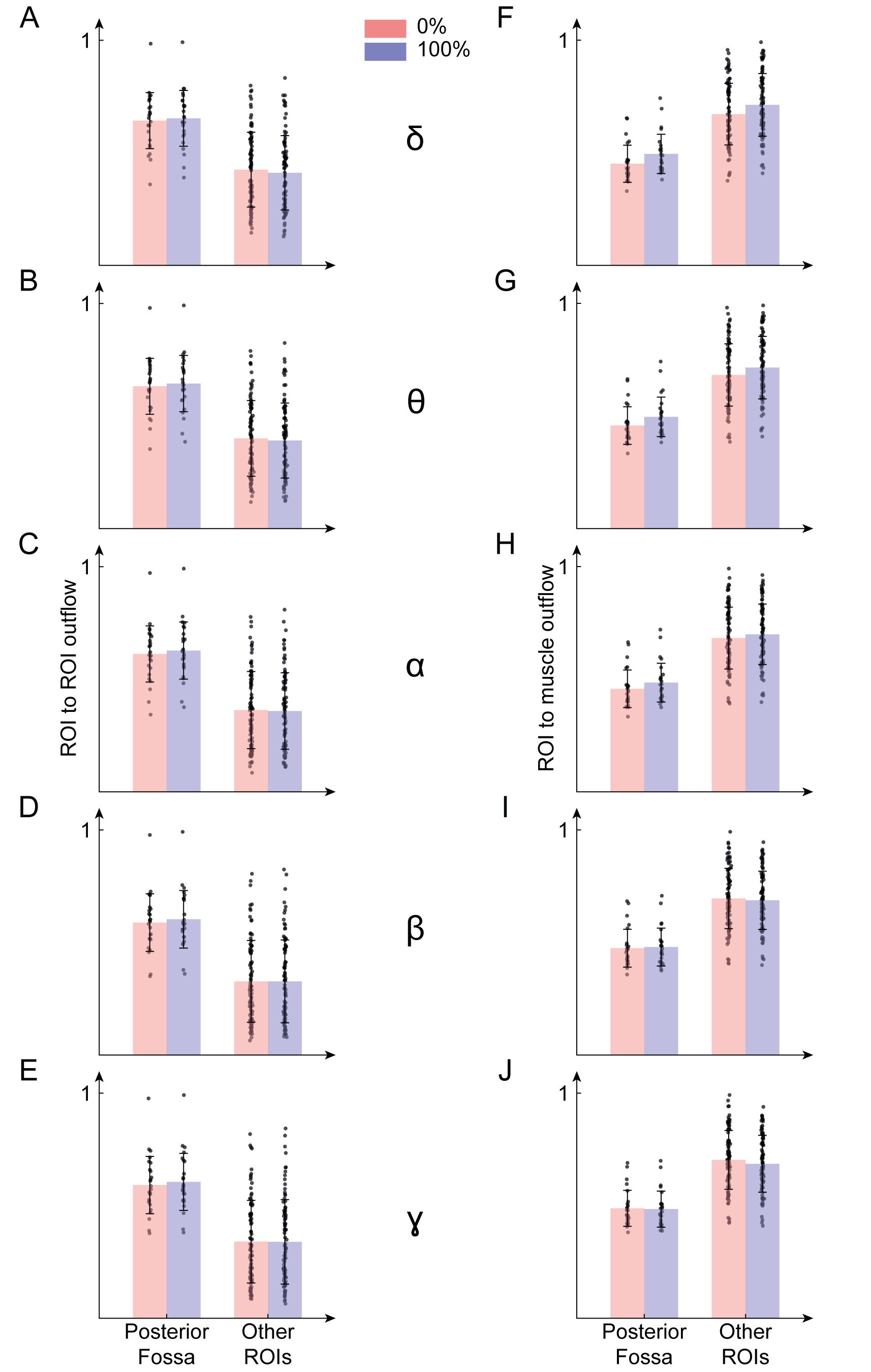


**Supplementary Figure 2.** **Posterior fossa vs. other ROIs in all frequency bands. (A)** to **(E)**  Normalized outflow of the ROIs of the posterior fossa in cortico-cortical connectivity and outflow of all other ROIs, for both guidance force levels, in each frequency band. **(F)** to **(J)** Normalized outflow of the ROIs of the posterior fossa in cortico-muscular connectivity and outflow of all other ROIs, for both guidance force levels, in each frequency band.
